# Supplementary material for: Developing a media formulation to sustain ex vivo chloroplast function
Source: Front Bioeng Biotechnol. 2025 Apr 9;13:1560200. doi: 10.3389/fbioe.2025.1560200 (PMC12014621; doi:10.3389/fbioe.2025.1560200)
Supplement: Supplementary file 1 [file DataSheet2.pdf]

**Supplementary table 2. Metabolite media formulations**

| Essential Metabolite Media | |
|---|---|
| **Metabolite** | **Concentration** |
| AMP | 1.2mM |
| ATP | 1.2mM |
| CMP | 1.2mM |
| CTP | 1.2mM |
| Mg2+ | 10mM |
| Histadine | 2mM |
| Alanine | 2mM |
| Arginine | 2mM |
| Asparagine | 2mM |
| Proline | 2mM |
| Aspartate | 2mM |

| Enhanced Essential Metabolite Media | |
|---|---|
| **Metabolite** | **Concentration** |
| ATP | 1.2mM |
| GMP | 0.86mM |
| UMP | 0.86mM |
| CMP | 0.86mM |
| E. coli tRNA | 170ug/mL |
| 19 Essential Amino Acids (excluding glutamate) | 2mM |
| NAD | 0.33mM |
| Coenzyme A | 0.27mM |
| Spermidine | 1.5mM |
| Putrescine | 1mM |
| Potassium oxalate | 2.7mM |
| Potassium glutamate | 175mM |
| Ammonium glutamate | 10mM |
| Magnesium glutamate | 10mM |
| PEP | 33mM |
