# Supplementary material for: Developing a media formulation to sustain ex vivo chloroplast function
Source: Front Bioeng Biotechnol. 2025 Apr 9;13:1560200. doi: 10.3389/fbioe.2025.1560200 (PMC12014621; doi:10.3389/fbioe.2025.1560200)
Supplement: Supplementary file 2 [file DataSheet1.pdf]

Supplementary table 1. List of essential reactions in chloroplasts

Note: The list reactions is specifically Chlamydomonas reinhardtii-related, however the model uses Nannochloropsis gaditana as a base, hence all reactions common to both organisms have a "Nano" designation.

Reaction ID	Chloroplast Essential Reactions
NanoG0117	'homoserine dehydrogenase'
NanoG0124	threonine synthase'
NanoG0130	'atp:l-homoserine o-phosphotransferase'
NanoG0131	'threonine ammonia-lyase'
NanoG0189	'aspartate---prephenate aminotransferase'
NanoG0191	'Chorismate synthase'
NanoG0193	'3-phosphoshikimate 1-carboxyvinyltransferase'
NanoG0199	'Phosphoribosylanthranilate isomerase'
NanoG0202	'3-dehydroquinate synthase'
NanoG0203	'shikimate kinase'
NanoG0204	'quinate/shikimate dehydrogenase'
NanoG0205	'anthranilate phosphoribosyltransferase'
NanoG0209	'anthranilate synthase'
NanoG0210	'3-dehydroquinate dehydratase'
NanoG0211	'3-deoxy-7-phosphoheptulonate synthase'
NanoG0258	'branched-chain-amino-acid transaminase'
NanoG0261	'acetolactate synthase'
NanoG0263	'ketol-acid reductoisomerase'
NanoG0276	'dihydroxy-acid dehydratase'
NanoG0281	'branched-chain-amino-acid transaminase'
NanoG0293	'4-hydroxy-tetrahydrodipicolinate synthase'
NanoG0324	'udp-sulfoquinovose synthase'
NanoG0348	'l-ascorbate peroxidase'
NanoG0350	'glutathione dehydrogenase (ascorbate)'
NanoG0401	'triose-phosphate isomerase'
NanoG0412	'phosphoglycerate kinase'
NanoG0514	'ribulose-phosphate 3-epimerase'
NanoG0516	'transketolase'
NanoG0528	'ribose-phosphate diphosphokinase'
NanoG0588	'phosphoribulokinase'
NanoG0589	'ribulose-bisphosphate carboxylase'
NanoG0609	'inorganic diphosphatase'
NanoG0677	'3-hydroxyacyl-[acyl-carrier-protein] dehydratase (n-c12:0)'
NanoG0679	'3-hydroxyacyl-[acyl-carrier-protein] dehydratase (n-c16:0)'
NanoG0681	'3-hydroxyacyl-[acyl-carrier-protein] dehydratase'
NanoG0683	'3-hydroxyacyl-[acyl-carrier-protein] dehydratase'
NanoG0685	'enoyl-[acyl-carrier-protein] reductase (nadph, re-specific)'
NanoG0688	'3-oxoacyl-[acyl-carrier-protein] reductase [(n-c4:0)]'
NanoG0690	'3-oxoacyl-[acyl-carrier-protein] reductase [(n-c6:0)]'
NanoG0692	'3-oxoacyl-[acyl-carrier-protein] reductase (n-c8:0)'
NanoG0694	'3-oxoacyl-[acyl-carrier-protein] reductase (n-c10:0)'
NanoG0696	'3-oxoacyl-[acyl-carrier-protein] reductase (n-c12:0)'
NanoG0698	'3-oxoacyl-[acyl-carrier-protein] reductase (n-c16:0)'
NanoG0699	'3-oxoacyl-[acyl-carrier-protein] reductase'
NanoG0704	'malonyl-coa:[acyl-carrier-protein] s-malonyltransferase/ malonyl-coa:acp-trans-acylase'
NanoG0712	'3-hydroxyacyl-[acyl-carrier-protein] dehydratase'
NanoG0714	'Acetyl transacylase'

NanoG0728	'stearoyl-[acyl-carrier-protein] delta9-desaturase ((9Z)-n-C18:1)'
NanoG0735	'enoyl-[acyl-carrier-protein] reductase (nadh)[n-c18:0]'
NanoG0736	'enoyl-[acyl-carrier-protein] reductase (nadh)'
NanoG0740	'enoyl-[acyl-carrier-protein] reductase (nadh)'
NanoG0742	'3-hydroxyacyl-[acyl-carrier-protein] dehydratase (n-c8:0)'
NanoG0744	'3-hydroxyacyl-[acyl-carrier-protein] dehydratase [(n-c6:0)]'
NanoG0746	'3-hydroxyacyl-[acyl-carrier-protein] dehydratase (n-c18:0)'
NanoG0755	'acyl-[acyl-carrier-protein] delta9-desaturase ((9Z)-n-C16:1)'
NanoG0771	'beta-ketoacyl-[acyl-carrier-protein] synthase i'
NanoG0785	'3-oxoacyl-[acyl-carrier-protein] synthase (C4:0 forming)'
NanoG0793	'3-oxoacyl-[acyl-carrier-protein] reductase (n-c18:0)'
NanoG0802	'acetyl-coa carboxylase'
NanoG1695	'galactolipid galactosyltransferase'
NanoG0892	'Phosphatidate phosphatase (n-C14 0)'
NanoG0894	'Phosphatidate phosphatase (n-C16 0)'
NanoG0896	'Phosphatidate phosphatase (n-C16 1)'
NanoG0898	'Phosphatidate phosphatase (n-C18 0)'
NanoG0900	'Phosphatidate phosphatase (n-C18 1)'
NanoG0902	'Phosphatidate phosphatase (n-C18 2)'
NanoG0904	'Phosphatidate phosphatase (n-C18 3)'
NanoG0908	'Phosphatidate phosphatase (n-C20 4)'
NanoG0910	'Phosphatidate phosphatase (n-C20 5)'
NanoG0911	'1-acylglycerol-3-phosphate O-acyltransferase (n-C14:0)'
NanoG0912	'1-acylglycerol-3-phosphate o-acyltransferase (n-C16:1)'
NanoG0913	'1-acylglycerol-3-phosphate o-acyltransferase (n-C18:0)'
NanoG0914	'1-acylglycerol-3-phosphate o-acyltransferase (n-C18:1)'
NanoG0923	'sulfoquinovosyltransferase (n-C16 0)'
NanoG0924	'sulfoquinovosyltransferase (n-C16 1)'
NanoG0925	'sulfoquinovosyltransferase (n-C18 1)'
NanoG0926	'sulfoquinovosyltransferase (n-C18 2)'
NanoG0927	'sulfoquinovosyltransferase (n-C20 4)'
NanoG0928	'sulfoquinovosyltransferase (n-C20 5)'
NanoG0959	'1-acylglycerol-3-phosphate o-acyltransferase (n-C16:0)'
NanoG0973	'UDPgalactose 1,2-diacylglycerol 3-beta-D-galactosyltransferase (n-C14 0)'
NanoG0975	'UDPgalactose 1,2-diacylglycerol 3-beta-D-galactosyltransferase (n-C16 0)'
NanoG0977	'UDPgalactose 1,2-diacylglycerol 3-beta-D-galactosyltransferase (n-C16 1)'
NanoG0979	'UDPgalactose 1,2-diacylglycerol 3-beta-D-galactosyltransferase (n-C18 1)'
NanoG0981	'UDPgalactose 1,2-diacylglycerol 3-beta-D-galactosyltransferase (n-C20 4)'
NanoG0983	'UDPgalactose 1,2-diacylglycerol 3-beta-D-galactosyltransferase (n-C20 5)'
NanoG0996	'glycerol-3-phosphate: acyl-coa acyltransferase C18:2'
NanoG0998	'glycerol-3-phosphate: acyl-coa acyltransferase C18:3'
NanoG1000	'glycerol-3-phosphate: acyl-coa acyltransferase C20:4'
NanoG1002	'glycerol-3-phosphate: acyl-coa acyltransferase C20:5'
NanoG1620	'sulfoquinovosyltransferase (n-C14 0)'
NanoG1631	'UDPgalactose 1,2-diacylglycerol 3-beta-D-galactosyltransferase (n-C18 2)'
NanoG1035	'glycerol-3-phosphate dehydrogenase [nad(p)+]'
NanoG1268	'glutamate-tRNA ligase'
NanoG1269	'Mg-protoporphyrin IX monomethyl ester (oxidative) cyclase I'
NanoG1270	'Mg-protoporphyrin IX monomethyl ester (oxidative) cyclase II'
NanoG1271	'Mg-protoporphyrin IX monomethyl ester (oxidative) cyclase III'
NanoG1273	'hydroxymethylbilane synthase'

NanoG1276	'magnesium protoporphyrin ix methyltransferase'
NanoG1278	'porphobilinogen synthase'
NanoG1279	'protochlorophyllide reductase'
NanoG1281	'chlorophyllide a oxygenase'
NanoG1282	'chlorphyll b reductase; chlorophyllide a oxygenase'
NanoG1283	'protoporphyrinogen oxidase'
NanoG1285	'glutamate-1-semialdehyde 2,1-aminomutase'
NanoG1289	'glutamyl-trna reductase'
NanoG1293	'coproporphyrinogen oxidase'
NanoG1294	'divinyl chlorophyllide a 8-vinyl-reductase'
NanoG1296	'chlorophyll synthase'
NanoG1302	'uroporphyrinogen decarboxylase'
NanoG1304	'uroporphyrinogen-iii synthase'
NanoG1310	'magnesium chelatase'
NanoG1338	'1-deoxy-d-xylulose-5-phosphate synthase'
NanoG1383	'glutathione-disulfide reductase'
NanoG1397	'lycopene cyclase (alpha-carotene producing)'
NanoG1398	'lycopene cyclase (delta-carotene producing)'
NanoG1399	'Neurosporene oxidoreductase'
NanoG1400	'phytoene desaturase (2)'
NanoG1404	'zeta-carotene desaturase'
NanoG1405	'alpha-carotene hydroxylase (alpha-cryptoxanthin forming)'
NanoG1408	'lycopene beta-cyclase'
NanoG1409	'lycopene beta-cyclase'
NanoG1414	'phytoene desaturase'
NanoG1423	'2-c-methyl-d-erythritol 4-phosphate cytidylyltransferase'
NanoG1424	'geranylgeranyl diphosphate reductase'
NanoG1425	'1-deoxy-d-xylulose-5-phosphate reductoisomerase'
NanoG1428	'(E)-4-hydroxy-3-methylbut-2-enyl-diphosphate synthase'
NanoG1429	'geranylgeranyl diphosphate synthase'
NanoG1434	'4-(cytidine 5''-diphospho)-2-c-methyl-d-erythritol kinase'
NanoG1435	'2-c-methyl-d-erythritol 2,4-cyclodiphosphate synthase'
NanoG1437	'(2e,6e)-farnesyl diphosphate synthase'
NanoG1439	'dimethylallyltranstransferase'
Tr_ADPHt_h	'ADP:H+ symporter, chloroplast'
Tr_AMETt2h_c	'S-Adenosyl-L-methionine reversible transport, chloroplast'
Tr_AMPt_h	'AMP transport, chloroplast'
Tr_ASPt_h	'Amino acid transporter (asp-L), chloroplast'
Tr_CMPt_h	'CMP transport via diffusion, chloroplast'
Tr_CTPt_h	'Plastid nucleotide transporter (CTP/ATP antiport)'
Tr_HISt_h	'Amino acid transporter (his-L), chloroplast'
Tr_MG2t_h	'Divalent cation (Mg2+) transport system, chloroplast'
Tr_UDPGALt_h	'UDP-galactose:UMP antiporter, chloroplast'
Tr_UDPGt_h	'UDP-glucose antiporter, chloroplast'
Tr_UDPt_h	'UDP transport via diffusion, chloroplast'
B_adp[c]	'B_adp[c]'
B_ahcys[c]	'B_ahcys[c]'
B_amet[c]	'B_amet[c]'
B_amp[c]	'B_amp[c]'
B_asp-l[c]	'B_asp-l[c]'
B_atp[c]	'B_atp[c]'

B_cmp[c]	'B_cmp[c]'
B_ctp[c]	'B_ctp[c]'
B_his-l[c]	'B_his-l[c]'
B_mg2[c]	'B_mg2[c]'
B_udpgal[c]	'B_udpgal[c]'
B_udpg[c]	'B_udpg[c]'
B_udp[c]	'B_udp[c]'
Ex_Photon	'Photon import'
PSII_Photon	'Directing photons to PSII'
PSI_Photon	'Directing photons to PSI'
S0S1	'Manganese cluster S-cycling, S0 -> S1'
S1S2	'Manganese cluster S-cycling, S1 -> S2'
S2S3	'Manganese cluster S-cycling, S2 -> S3'
S3S4	'Manganese cluster S-cycling, S3 -> S4'
S4S0	'Manganese cluster S-cycling, S4 -> S0'
P680P	'Electron transport P680 -> Pheophytin a'
YZP680	'Electron transport Yz -> P680'
PQA	'Electron transport P680 -> QA'
QAQB1	'First reduction of QB'
QAQB2	'Second reduction of QB'
QBPQH2	'QB enters the PQ pool'
PQPSII	'Docking of PQ to PSII -> QB(ox)'
PQH2R	'Reduction of Rieske ISP in cyt b6f complex'
RISPCF	'Electron transfer from Rieske ISP to Cyt f'
CFPC	'Electron transfer from Cyt f to PC'
PQRHBP	'Electron transfer from PQ radical to Heme bp'
HBPHBNHCN1	'First reduction of Heme bn/Heme cn by Heme bp'
HBPHBNHCN2	'Second reduction of Heme bn/Heme cn by Heme bp'
HBNHCNPQH2	'Re-oxidation of Heme bn/Heme cn and generation of secondary PQH2'
P700A0	'Excitation of P700'
PCP700	'Re-reduction of P700 by PC'
A0A1	'Electron transfer from A0 to A1'
A1FX	'Electron transfer from A1 to Fx'
FXFB	'Electron transfer from Fx to FB'
FBFA	'Electron transfer from FB to FA'
FAFD	'Electron transfer from FA to Ferredoxin'
FDFNR1	'First reduction of FNR'
FDFNR2	'Second reduction of FNR'
FNRNADPH	'Reduction of NADP+ by FNR'
ATPSh	'ATP synthase'
Tr_H2O_u	'H2O transport, thykaloid'
Tr_O2_u	'O2 transport, thykaloid'
ala-l[h]	'L-alanine import to chloroplast'
B_ala-l[c]	'B_ala-l[c]'
arg-l[h]	'L-Arginine import to chloroplast'
B_arg-l[c]	'B_arg-l[c]'
asn-l[h]	'L-Asparagine chloroplast import'
B_asn-l[c]	'B_asn-l[c]'
Tr_pro_h	'L-Proline transport c <-> h'
B_pro-l[c]	'B_pro-l[c]'
R04199_h	'2,3,4,5-tetrahydrodipicolinate:NADP+ 4-oxidoreductase'

R07613_h	'LL-2,6-diaminoheptanedioate:2-oxoglutarate aminotransferase'
R02735_h	'LL-2,6-Diaminoheptanedioate 2-epimerase'
R00451_h	'meso-2,6-diaminoheptanedioate carboxy-lyase'
R00650_h	'Homocysteine S-methyltransferase'
R00691_h	'Arogenate dehydratase'
R00732_h	'Arogenate dehydrogenase'
R01213_h	'Isopropylmalate synthase'
R10170_h	'Isopropylmalate isomerase'
R04426_h	'Isopropylmalate dehydrogenase'
R01652_h	'2-Oxoisocaproate synthesis (spontaneous)'
R01090_h	'Branched-chain aminotransferase'
@Chl_protprod	'Chlamydomonas chloroplast protein production'
R06946_h	'Zeaxanthin epoxidase'
R06947_h	'Zeaxanthin epoxidase'
R06948_h	'Neoxanthin synthase'
R01195_h	'Ferredoxin:NADP+ oxidoreductase'
B_c182coa[c]	'B_c182coa[c]'
c182coa_h	'C18:2-CoA transport c -> h'
R02241_c182_h	'Acyl-CoA:1-acyl-sn-glycerol-3-phosphate 2-O-acyltransferase'
B_c183coa[c]	'B_c183coa[c]'
c183coa_h	'C18:3-CoA transport c -> h'
R02241_c183_h	'Acyl-CoA:1-acyl-sn-glycerol-3-phosphate 2-O-acyltransferase'
B_c204(6)coa[c]	'B_c204(6)coa[c]'
c204(6)coa_h	'C20:4(6)-CoA transport c -> h'
R02241_c204_h	'Acyl-CoA:1-acyl-sn-glycerol-3-phosphate 2-O-acyltransferase'
B_c205(3)coa[c]	'B_c205(3)coa[c]'
c205(3)coa_h	'C20:5(3)-CoA transport c -> h'
R02241_c205_h	'Acyl-CoA:1-acyl-sn-glycerol-3-phosphate 2-O-acyltransferase'
_@Chl@Pha_dhacoa[	'B_@C@P_dhacoa[c]'
Chl@Pha_Tr_dhacoa	'DHA transport c -> h (Phaeo and Chlamy specific rx)'
@Chl_dhag3psynth	'Glycerol-3-phosphate:acyl-coa acyltransferase C22:6 (Chlamy specific)'
@Chl_R02241_c226_	'Acyl-CoA:1-acyl-sn-glycerol-3-phosphate 2-O-acyltransferase (Chlamy specific)'
@Chl_dg226synth	'Phosphatidate phosphatase (n-C22:6) (Chlamy specific)'
@Chl_mgdg226synth	'UDPgalactose 1,2-diacylglycerol 3-beta-D-galactosyltransferase (n-C22:6) (Chlamy specific)'
@Chl_dgdg226synth	'Galactolipid galactosyltransferase (Chlamy specific)'
@Chl_sqdg226synth	'Sulfoquinovosyltransferase (n-C22:6) (Chlamy specific)'
mgdg180synth	'UDPgalactose 1,2-diacylglycerol 3-beta-D-galactosyltransferase (n-C18)'
dgdg180synth	'Galactolipid galactosyltransferase'
sqdg180synth	'Sulfoquinovosyltransferase (n-C18)'
@Chl_mgdg183synth	'UDPgalactose 1,2-diacylglycerol 3-beta-D-galactosyltransferase (n-C18 3)'
@Chl_dgdg183synth	'Galactolipid galactosyltransferase'
@Chl_sqdg183synth	'Sulfoquinovosyltransferase (n-C18 3)'
@Chl_mgdg_pf	'Chlamydomonas MGDG pool formation'
@Chl_dgdg_pf	'Chlamydomonas DGDG pool formation'
@Chl_sqdg_pf	'Chlamydomonas SQDG pool formation'
@Chl_ccmemprod	'Chlamydomonas chloroplast membrane lipid pool formation'
@Chl_R09067_h	'Chlorophyllide-b:phytyl-diphosphate phytyltransferase'
@Chl_R07851_h	'Alpha-cryptoxanthin,reduced ferredoxin [iron-sulfur] cluster:oxygen 3-oxidoreductase'
@Chl_pigm	'Chlamydomonas pigment fraction assembly'
@Chl_bio	'Chlamydomonas chloroplast biomass'
